# Knowledge and Attitudes Toward Healthcare Services Among a Particularly Vulnerable Tribal Group (PVTG) in Coastal Karnataka, India: Findings from a Community-Based Cross-Sectional Survey

**DOI:** 10.12688/f1000research.176835.1

**Published:** 2026-04-16

**Authors:** Biju Soman, Sneha D Mallya, Rishi S Narayanan, Vani Lakshmi R., Ashwini Kumar, B. Unnikrishnan, Harpreet Kaur, Ranjitha S Shetty

**Affiliations:** 1 Department of Community Medicine, Kasturba Medical College, Manipal Academy of Higher Education, Manipal, India; 2 Centre for Digital Health, Applied Research and Technology, Prasanna School of Public Health, Manipal Academy of Higher Education, Manipal, India; 3 Department of Community Medicine, Kasturba Medical College, Mangalore, Manipal Academy of Higher Education, Manipal, India; 4 Division of Epidemiology and Communicable Diseases, Ministry of Health and Family Welfare, Indian Council of Medical Research, New Delhi, Delhi, 110029, India

**Keywords:** Indigenous Health Services, Tribal, Knowledge, Attitude, India, Traditional Medicine

## Abstract

**Background:**

Among various Particularly Vulnerable Tribal Groups (PVTGs) in India, the Koraga community stands out as one that warrants focused attention through targeted public health efforts.

**Objective:**

A community-based cross-sectional study was conducted to assess knowledge and attitudes toward healthcare services among 622 adult members (aged 18–70 years) of the Koraga tribe from a southern coastal district of Karnataka.

**Methods:**

We employed a multistage cluster sampling approach, treating each block under the study as a cluster and selecting households using probability proportional to size (PPS) method.

Data collection survey occurred from April 2021 to April 2022 through personal interviews with consenting adults, using a structured data collection tool.The data was entered and analyzed using Jamovi software version 2.6.26.

Univariate and multivariable logistic regression was performed to examine associations between sociodemographic variables and the knowledge and attitude for communicable diseases, reproductive health, and mental health services.

The results are represented as unadjusted odds ratio and adjusted odds ratio with 95% confidence intervals.

**Results:**

The study found that, overall, 58% of respondents exhibited poor knowledge as well as unfavourable attitudes toward utilising healthcare services.

The study revealed limited knowledge among tribal people regarding healthcare services, with scores of 62.2% for communicable diseases, 71.9% for reproductive health, and 50% for mental health. Additionally, attitudes toward these domains were largely unfavourable, measured at 50.2%, 54.8%, and 60.5%, respectively.

These outcomes showed a statistically significant association with demographic variables, including head-of-household status, age, religion, family size, educational attainment, and residence (p < 0.05).

**Conclusions:**

PVTG community in Udupi district of South Karnataka, India, lack knowledge and favourable attitude towards healthcare services on communicable diseases, reproductive health and mental health. The findings underscore the urgent need for tailored health literacy initiatives and follow-up interventions among this PVTG to improve their health beliefs, and health status.

## Introduction

Tribal communities in India are diverse and unique with each having its own distinct cultural and social characteristics. They comprise various ethno-linguistic groups, each with distinct beliefs and differing levels of economic, educational, and cultural development. ^1^ Approximately 8.6% of India’s population, is constituted by tribals with largest concentrations of Scheduled Tribes (ST) being found in states such as Andhra Pradesh, Assam, Jharkhand, Gujarat, Chhattisgarh, Maharashtra, Odisha, Rajasthan, and West Bengal. Almost 94.43 per cent of the total population in Mizoram and 94.79 per cent in Lakshadweep are Scheduled Tribes (STs). ^2^

Research studies focusing on indigenous populations have identified several significant health issues, including malaria, sexually transmitted infections (STIs), tuberculosis, nutritional deficiencies, and genetic disorders such as glucose-6-phosphate dehydrogenase (G6PD) deficiency and sickle cell anaemia. ^3, 4^ In India, tribal healthcare is typically categorized under rural healthcare. However, it is a misconception to assume that the problems and needs of tribal populations mirror those of rural communities. ^5^ The unique geographical, environmental, social, and cultural contexts of tribal groups result in distinct healthcare requirements. These communities are profoundly affected by socio-cultural and economic factors, and their limited access to healthcare services, exacerbated by their widespread distribution, renders them particularly vulnerable within the framework of India’s National Strategic Plan. ^6, 7^ Further, the tribal people in India have poorest health status contributed by complete absence of community participation in the planning, design and implementation of health services.

There are about 42,48,978 tribal people in Karnataka, a southern coastal state in India, which accounts to 6.95% of the total population of the state. The ST population of the state is primarily rural (84.7%). Among the major STs, *Koli Dhor* have the highest (92.2%) rural population, followed by *Gond* (91.7%), *Marati* (90.8%) and *Naika* (85.1%). District-wise distribution of ST population shows that the tribal population is present in all 31 districts of Karnataka state with eight districts accounting for 52% of the tribal population of the state. There are two Particularly Vulnerable Tribal Groups (PVTG) in Karnataka, and they are *Koraga and Jenukuruba* tribes. ^8^

The problems and health issues of Koraga community are often overlooked, though the health issues of this tribal group are many. Magnitude of alcohol dependence was considerably more among Koraga tribe with a positive family history of alcoholism compared to other tribal groups and general population. ^9^ Prior published research findings reiterate that gender sensitive interventions and strengthening of the health systems should be emphasised in India, to improve health status and utilization of healthcare services by tribal communities. ^10^ Very few studies are done on the healthcare delivery system among tribal groups. Previous studies have demonstrated that prevailing health beliefs and practices among tribal communities significantly influence their access to and utilization of healthcare services. These findings underscore the urgent need to raise awareness and reduce barriers to healthcare utilization through targeted education. ^11– 14^

There is limited research assessing the knowledge, attitudes, and practices related to the healthcare delivery system among tribal populations. Considering the paucity of research on this subject among this PVTG, the present community-based study was undertaken to assess the knowledge and attitudes of the Koraga community residing in coastal Karnataka towards healthcare services. ^15– 18^ This research aligns with Sustainable Development Goals 3, 4 and 10 (good health and well-being, quality education and reduced inequalities).

## Materials and methods

In Udupi district, located in southern part of India, the Koraga community is distributed across seven blocks. There are a total of 2653 Koraga households in Udupi district comprising a population of 8648 individuals. (Source: Integrated Tribal Development Project (ITDP) office, Udupi District).

A community-based, cross-sectional survey was conducted among the Koraga tribe across all seven blocks of Udupi district from April 2021 to April 2022. Udupi, a coastal district in Karnataka, features diverse geographical terrain, including forests, hills, rivers, and extensive seashores. Many of the tribal families are concentrated in forested and remote areas, where access to mainstream society is limited. A sample size of 622 was estimated based on results of a pilot study showing 50% of the tribal population to have knowledge on healthcare services and its utilization, absolute precision of 5%, design effect of 1.5, at 5% level of significance and non-response of 10% by applying the finite population correction. The list of households and tribal hamlets residing in each of the seven blocks was obtained from ITDP office and multistage cluster sampling was used to identify the households. Each block was considered as a cluster and the number of households that need to be included was calculated based on probability proportional to size (PPS) sampling method. ^19^

**Figure 1.**
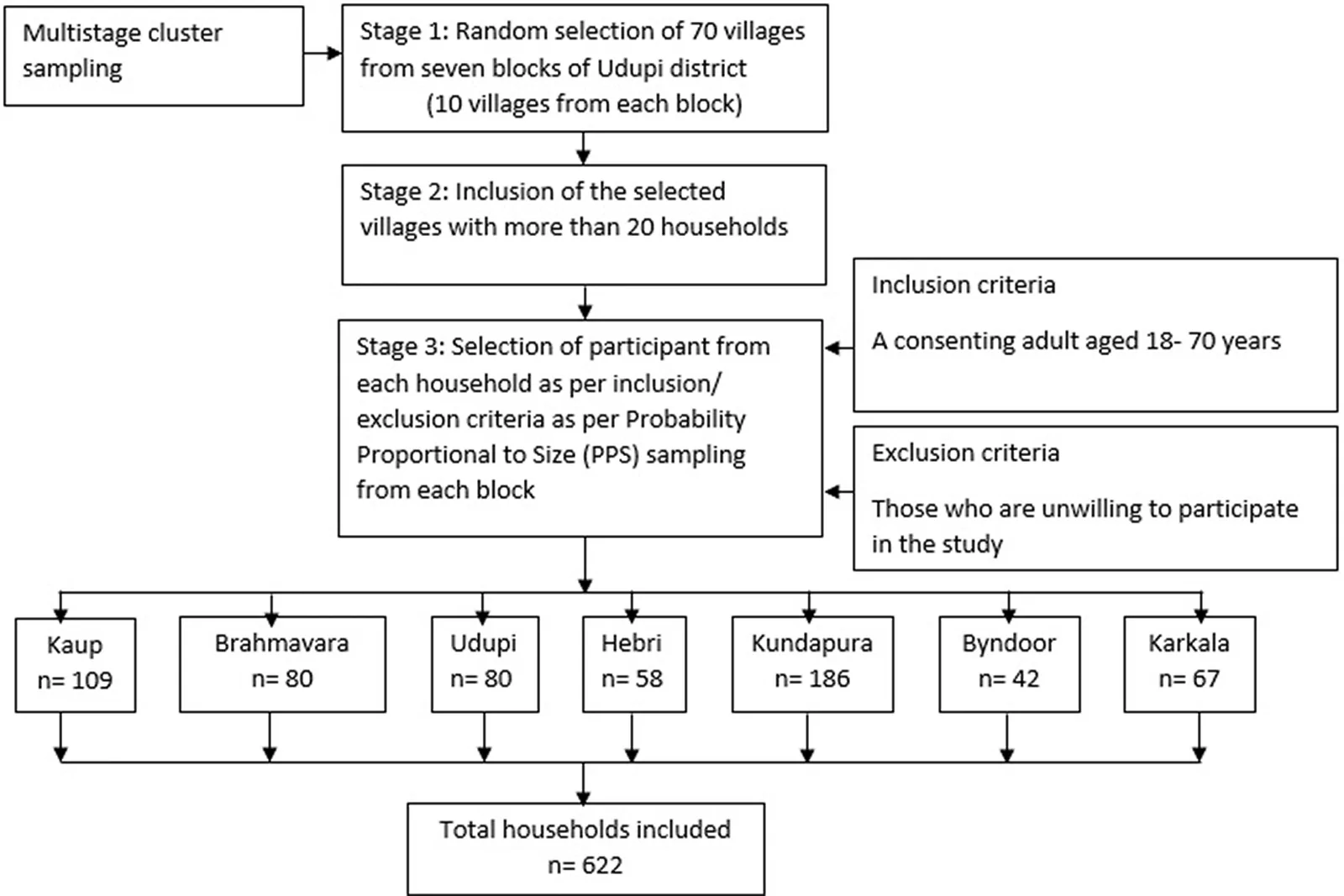
STROBE Flow diagram. The STROBE flow diagram depicts the probability proportional to size (PPS) sampling method to select the households included in the study.

Approximately 70 villages were selected randomly from all seven blocks, and a minimum of 20 households were selected from each village. Consenting family members aged 18–70 years were included in the survey. Those who were unwilling to participate in the survey were excluded ( Figure 1). The study was carried out, with necessary administrative and technical support from the Udupi district administration, ITDP, Udupi district, District Health and Family Welfare Department and Samagra Grameena Ashram, a non-governmental organization (NGO), functioning in the Udupi district, and tribal community gate keepers.

The data was collected by the research survey team, including a research assistant and a Ph.D. scholar in health sciences who were trained in collecting data from the tribal community by the Principal Investigator (PI). Selected men and women volunteers from the tribal community as well as grassroot level health care workers namely, Accredited Social Health Activists (ASHA), catering to tribal population helped in data collection process by facilitating the introduction of survey team and briefing about the research project. Interviews were conducted at the households after explaining the purpose of survey and a detailed participant information sheet (PIS) was provided to the consented healthy male or female participants at their households. A written informed consent (IC) was obtained from the participants, and they were informed that their details would be kept confidential. All the ethical guidelines in line with declaration of Helsinki were followed during the conduct of the study. ^20^ The questionnaire used for the study consisted of two sections and was validated by experts in community medicine and public health and was pretested among ten tribal members and found to be reliable and feasible. The interviewer administered questionnaire included details on demographic particulars, and knowledge and attitude questions pertaining to the health care system and services across three domains including communicable diseases, reproductive health and mental health. The questionnaire consisted of a total of 35 questions: communicable diseases [knowledge:6; attitude:8], reproductive health [knowledge:5; attitude:5] and mental health [knowledge:6; attitude:5]. The knowledge questions were scored from 0 to 1, with a correct answer given 1 point and an incorrect answer being 0. The attitude questions, which were on a Likert scale, were given points from 0 to 1, with 0 for options suggesting an unfavorable attitude and 1 point for a favorable attitude. The range of scores for knowledge and attitude on communicable diseases was 0–6 and 0–8, respectively. Similarly, the knowledge and attitude scores for reproductive health were 0–5 and 0–5, respectively. Further, the knowledge and attitude scores for mental health were 0–6 and 0–5 respectively. The median scores were calculated for knowledge and attitude for all three domains. An individual was considered to have adequate knowledge and a favorable attitude for a given domain if the scores were above the median for the given domain. Each participant took about 20–25 minutes to answer the questions, which were asked in the local language Kannada and the responses were recorded in the data collection sheet by the researchers. Since the interviews were conducted by trained researchers and a structured questionnaire was used for data collection, there was no missing data.

### Ethics statement

The study was initiated after obtaining approval from Kasturba Medical College Kasturba Hospital Institutional Ethics Committee (IEC No- 778/2019). This study was registered under CTRI, with registration number CTRI/2019/12/022385. Permission was obtained from the District Health and Family Welfare Department, Integrated Tribal Development Project (ITDP) Udupi district and District Administrative Authority, Udupi district. The purpose of the study and data collection plan was discussed with the tribal community leaders. Queries and clarifications sought by them were clarified promptly by the research team, and they were assured about the anonymity and confidentiality of the information obtained during data collection.

### Statistical analysis

The data was entered and analyzed using Jamovi *software (version 2.6.26).* The categorical variables are presented as frequency and percentage. Univariate and multivariable logistic regression analysis was done to identify association between adequacy of knowledge and attitude towards communicable diseases, reproductive health and mental health and various background characteristics and unadjusted and adjusted odds ratio (UOR and AOR) was calculated and has been reported with 95% confidence interval. Variables which were significant on univariate analysis were included in the multivariable analysis. A p value <0.05 was considered statistically significant.

## Results

The present community-based study included a total of 622 Koraga tribal individuals with a female preponderance (64.8%). Table 1 depicts the background characteristics of the study participants. Among the 622 study participants, 73.6% of the participants were aged ≤60 years. Most of the participants (29.9%) were from Kundapura block, and out of all the households in the study, 35.7% of them had more than four members in the family. However, 63.2% of the study participants were from a nuclear family. Almost half (45.8%) of the respondents had only primary level of education, while 33% were illiterate. About 68.8% of the participants had the availability of a public healthcare facility within five kilometres, and 58.8% of the study population depended on public transport to reach it. Majority of the respondents (93.1%) reported that ASHAs visit their homes regularly at least once a month.

**Table 1.** Background characteristics of the study participants (n = 622).

Characteristics	Frequency
	n (%)
Head of the household	
Males	219 (35.2)
Females	403 **(64.8)**
Age group (in years)	
18–59	458 **(73.6)**
>60	164 (26.4)
Block	
Udupi	80 (12.9)
Kaup	109 (17.5)
Kundapura	186 **(29.9)**
Karkala	67 (10.8)
Brahmavara	80 (12.9)
Byndoor	42 (6.8)
Hebri	58 (9.3)
Total number of family members	
2	65 (10.5)
3	147 (23.6)
4	188 (30.2)
>4	222 **(35.7)**
Type of family	
Nuclear	393 **(63.2)**
Joint	183 (29.4)
Extended	46 (7.4)
Religion	
Hindu	260 (41.8)
Adidharma *	362 **(58.2)**
Educational status	
Illiterate	205 (33.0)
Primary school	285 **(45.8)**
Secondary school	92 (14.8)
Preuniversity college (PUC) and above	40 (6.4)
Distance from the nearest healthcare facility (in kilometres)	
< 5	422 **(67.8)**
6–10	126 (20.3)
>10	74 (11.9)
Mode of transport used to visit the healthcare facility	
Public transport	366 **(58.8)**
Private vehicle	256 (41.2)
Monthly home visits by ASHAs	
Yes	579 **(93.1)**
No	43 (6.9)

* ***Adidharma*** *, is a distinct religion of the tribal community in India, who believe in nature spirits and supreme power of nature, and does not practice idol worship.*

Table 2 shows that more than half of respondents had inadequate knowledge (62.2%) and an unfavorable attitude (50.2%) towards prevention and management of communicable diseases, while it was found to be even higher for reproductive health (71.9% and 54.8%). However, 50% of them had adequate knowledge on mental health, while 60.5%, had an unfavorable attitude towards mental illness and its treatment, which was very high compared to the other two domains. The item-wise table is provided in extended data. ( https://figshare.com/s/e4d0b21a90efc9e46b78).

**Table 2.** Domain-wise knowledge and attitude of the participants (n = 622).

**Knowledge domains**	**Frequency (%)**
***Communicable diseases***	
Adequate	235 (37.8)
Inadequate	387 (62.2)
***Reproductive health***	
Adequate	175 (28.1)
Inadequate	447 (71.9)
***Mental Health***	
Adequate	311 (50.0)
Inadequate	311 (50.0)
**Attitude domains**	
***Communicable diseases***	
Favorable	310 (49.8)
Unfavorable	312 (50.2)
***Reproductive health***	
Favorable	281 (45.2)
Unfavorable	340 (54.8)
***Mental health***	
Favorable	246 (39.5)
Unfavorable	376 (60.5)

*The major domains included in this study were communicable diseases, reproductive health and mental health, based on the data available from literature review and discussion with subject experts in the field.*

Table 3 depicts, on univariate analysis, all the factors except gender of the head of the household, total number of family members residing and type of family were found to be significantly associated with inadequate knowledge on communicable diseases (p<0.05). Further, on multivariable analysis, those with inadequate knowledge on communicable diseases were more likely to be aged above 60 years (AOR= 2.12; 95% CI 1.34-3.37), residents of Udupi (AOR=6.23, 95% CI 2.60-15.00), Kaup (AOR= 6.17, 95% CI 2.81-13.54), Kundapura (AOR=2.77, 95% CI 1.41- 5.43) and Karkala block (AOR= 2.74, 95% CI 1.27-5.88), from nuclear families (AOR= 2.27, 95% CI 1.04-4.94), following Adidharma religion (AOR= 1.75, 95% CI 1.14-2.67). Similarly, literacy level was also found to be a predictor for inadequate knowledge on communicable diseases with illiteracy (AOR=4.91, 95% CI 2.22-10.80), primary school (AOR=3.02, 95% CI 1.44-6.34) and secondary school educational status (AOR= 2.55, 95% CI 1.11-5.86), showing a significant association.

**Table 3.** Association between knowledge and attitudes toward healthcare services for communicable diseases and the demographic variables of the study population (n = 622).

Socio-demographic Characteristics	Category	Knowledge towards Healthcare Services for Communicable Diseases					
		Adequate n = 235 No. (%)	Inadequate n = 387 No. (%)	UOR (95% CI)	p value	AOR (95% CI)	p value
Head of the household	Male	69 (39.5)	150 (68.5)	0.66 (0.47–0.93)	0.017	1.37 (0.93–2.01)	0.11
	Female	166 (41.2)	237 (58.8)	1			
Age	≤60 years	199 (43.4)	259 (56.6)	1			
	>60 years	36 (22.0)	128 (78.0)	2.73 (1.81–4.13)	<0.001	2.12 (1.33–3.37)	<0.001*
Block	Udupi	16 (20.0)	64 (80.0)	8.21 (3.79–17.82)	<0.001	6.23 (2.60–14.94)	<0.001*
	Kaup	22 (20.2)	87 (79.8)	8.11 (3.95–16.69)	<0.001	6.17 (2.80–13.55)	<0.001*
	Kundapura	71 (38.2)	115 (61.8)	3.32 (1.79–6.20)	<0.001	2.77 (1.41–5.43)	0.003
	Karkala	27 (40.3)	40 (59.7)	3.04 (1.46–6.34)	0.003	2.73 (1.28–5.89)	0.010
	Brahmavara	37 (46.3)	43 (53.8)	2.39 (1.18–4.81)	0.015	2.02 (0.95–4.32)	0.068
	Byndoor	23 (54.8)	19 (45.2)	1.70 (0.75–3.84)	0.206	1.41 (0.59–3.40)	0.440
	Hebri	39 (67.2)	19 (32.8)	1			
Total family members	Two	25 (38.5)	40 (61.5)	1			
	Three	57 (38.8)	90 (61.2)	0.99 (0.54–1.80)	0.965		
	Four	74 (39.4)	114 (60.6)	0.96 (0.54–1.72)	0.898		
	>Four	79 (35.6)	143 (64.4)	1.13 (0.64–2.00)	0.671		
Type of Family	Nuclear	159 (40.5)	234 (59.5)	1			
	Joint	66 (36.1)	117 (63.9)	1.20 (0.83–1.73)	0.315	1.01 (0.67–1.54)	0.934
	Extended	10 (21.7)	36 (78.3)	2.45 (1.19–5.07)	0.016	2.27 (1.04–4.94)	0.039
Religion	Hindu	128 (49.2)	132 (50.8)	1			
	Adidharma	107 (29.6)	255 (70.4)	2.31 (1.66–3.22)	<0.001	1.74 (1.14–2.67)	0.010*
Educational status	Illiterate	59 (28.8)	146 (71.2)	0.67 (0.35–1.25)	0.209	4.91 (2.22–10.80)	<0.001*
	Primary school	117 (41.1)	168 (58.9)	2.15 (1.01–4.23)	0.026	3.08 (1.43–6.34)	0.004
	Secondary school	35 (38.0)	57 (62.0)	2.44 (1.14–5.22)	0.021	2.55 (1.11–5.86)	0.027
	PUC and above	24 (60.0)	16 (40.0)	1			

Socio-demographic Characteristics	Category	Attitude towards Healthcare Services for Communicable Diseases					
		Favorable n = 310 No. (%)	Unfavorable n = 312 No. (%)	C OR (95% CI)	p value	AOR (95% CI)	p value
Head of the household	Male	111 (50.7)	108 (49.3)	1.05 (0.76–1.46)	0.756		
	Female	199 (49.4)	204 (50.6)	1			
Age	≤60 years	245 (53.5)	213 (46.5)	1			
	>60 years	65 (39.6)	99 (60.4)	1.75 (1.22–2.52)	0.002	1.27 (0.83–1.96)	0.273
Block	Udupi	35 (43.8)	45 (56.3)	8.03 (3.38–19.12)	<0.001	3.62 (1.41–9.33)	0.008*
	Kaup	32 (29.4)	77 (70.6)	15.03 (6.41–35.28)	<0.001	8.80 (3.54–21.88)	<0.001*
	Kundapura	108 (58.1)	78 (41.9)	4.51 (2.03–10.06)	<0.001	3.16 (1.35–7.44)	0.008*
	Karkala	36 (53.7)	31 (46.3)	5.38 (2.21–3.07)	<0.001	5.78 (2.29–14.59)	<0.001*
	Brahmavara	36 (45.6)	44 (55.6)	7.63 (3.21–18.17)	<0.001	4.95 (1.97–12.44)	<0.001*
	Byndoor	13 (31.0)	29 (69.0)	13.94 (5.17–37.61)	<0.001	11.87 (4.13–34.07)	<0.001*
	Hebri	50 (86.2)	8 (13.8)	1			
Total family members	Two	32 (49.2)	33 (50.8)	1			
	Three	79 (53.7)	68 (46.3)	0.84 (0.47–1.50)	0.544		
	Four	90 (47.9)	98 (52.1)	1.06 (0.60–1.86)	0.850		
	>Four	109 (49.1)	113 (50.9)	1.01 (0.58–1.75)	0.985		
Type of family	Nuclear	208 (52.9)	185 (47.1)	1			
	Joint	80 (43.7)	103 (56.3)	1.45 (1.02–2.06)	0.040	1.22 (0.81–1.83)	0.351
	Extended	22 (47.8)	24 (52.2)	1.23 (0.67–2.26)	0.513	1.16 (0.58–2.33)	0.672
Religion	Hindu	178 (68.5)	82 (31.5)	1			
	Adidharma	132 (36.5)	230 (63.5)	3.78 (2.70–5.30)	<0.001	4.06 (2.62–6.28)	<0.001*
Educational status	Illiterate	77 (37.6)	128 (62.4)	2.03 (1.02–4.03)	0.042	2.91 (1.33–6.34)	0.007*
	Primary school	162 (56.8)	123 (43.2)	0.92 (0.48–1.81)	0.826	1.20 (0.58–2.50)	0.624
	Secondary school	49 (53.3)	43 (46.7)	1.07 (0.51–2.26)	0.854	0.99 (0.44–2.24)	0.984
	>10 years of schooling	22 (55.0)	18 (45.0)	1			

**p* < 0.05 indicates statistically significant association*.

Educational status, religion and block had positive association with knowledge on services towards communicable diseases, while educational status, religion and block had positive association with attitudes towards the same domain.

Further, on univariate analysis, unfavorable attitude towards communicable diseases was significantly associated with age, block, type of family, religion and educational status (p<0.05). However, on multivariable analysis, respondents from Udupi (AOR= 3.63, 95% CI 1.41-9.34), Kaup (AOR= 8.80, 95% CI 3.54-21.88) Kundapura (AOR= 3.16, 95% CI 91.35-7.44), Karkala (AOR=5.78, 95% CI 2.29-14.59), Brahmavara ( AOR= 4.95, 95% CI 1.97-12.44) and Byndoor block (AOR= 11.87, 95% CI 4.13-34.07), were more likely to have unfavorable attitude as compared to those from Hebri block. Likewise, participants from joint families (AOR= 1.22, 95% CI 0.81-1.83), Adidharma religion (AOR= 4.06, 95% CI 2.63- 6.28) and those with low literacy (AOR= 2.91, 95% CI 1.33-6.34) also had a significant association with unfavorable attitude towards prevention and control of communicable diseases.

Table 4 shows in the domain of knowledge regarding reproductive health care services, on univariate analysis, there was a significant association with demographic characteristics such as age, block and religion (p < 0.05). Further, multivariable logistic regression analysis showed that those with inadequate knowledge of reproductive health were more likely to be aged above 60 years (AOR = 2.75, 95% CI 1.69–4.48), residents of Kaup block (AOR = 2.59, 95% CI 1.11–6.06) and follow Adidharma religion (AOR = 2.76, 95% CI 1.79–4.26). The univariate analysis between demographic variables and attitude towards reproductive healthcare services showed that there was a significant association with all the demographic variables other than total number of family members and educational status (p < 0.05). On multivariable analysis, those above 60 years (AOR = 1.82, 95% CI 1.18–2.79), residents of Udupi (AOR = 11.22, 95% CI 4.06–30.10), Kaup (AOR = 15.03, 95% CI 5.75–39.29), Kundapura (AOR = 5.24, 95% CI 2.18–12.59), Karkala (AOR = 7.69, 95% CI 2.96–19.10) and Byndoor blocks (AOR = 17.99, 95% CI 6.05–53.54), joint family (AOR = 1.72, 95% CI 1.12–2.66) and Adidharma religion (AOR = 3.18, 95% CI 2.06–4.91) were predictors of unfavourable attitude towards reproductive healthcare services compared to those from Hebri block and Hindu religion.

**Table 4.** Association between knowledge and attitude towards healthcare services on reproductive health with demographic variables of the study population (n = 622).

Socio-demographic Characteristics	Category	Knowledge towards Reproductive Healthcare Services					
		Adequate n = 175 No. (%)	Inadequate n = 447 No. (%)	C OR (95% CI)	*p* value	AOR (95% CI)	*p* value
Head of the household	Male	48 (21.9)	171 (78.1)	0.61 (0.42–0.90)	0.011	1.371 (0.90–2.07)	0.133
	Female	127 (31.5)	276 (68.5)	1			
Age	≤60 years	150 (32.8)	308 (67.2)	1			
	>60 years	25 (15.2)	139 (84.8)	2.71 (1.70–4.33)	<0.001	2.75 (1.69–4.48)	<0.001*
Block	Udupi	16 (20.0)	64 (80.0)	2.44 (1.14–5.24)	0.022	0.97 (0.41–2.27)	0.937
	Kapu	12 (11.0)	97 (89.0)	4.94 (2.21–1.00)	<0.001	2.59 (1.10–6.06)	0.028*
	Kundapura	62 (33.3)	124 (66.7)	1.22 (0.66–2.25)	0.520	0.72 (0.34–1.40)	0.343
	Karkala	21 (31.3)	46 (68.7)	1.33 (0.63–2.81)	0.440	1.31 (0.61–2.81)	0.488
	Brahmavara	34 (42.5)	46 (57.5)	0.82 (0.41–1.65)	0.590	0.45 (0.23–1.05)	0.066
	Byndoor	8 (19.0)	34 (81.0)	2.60 (1.01–6.62)	0.045	1.90 (0.72–5.02)	0.193
	Hebri	22 (37.9)	36 (62.1)	1			
Total family members	Two	17 (26.2)	48 (73.8)	1			
	Three	43 (29.3)	104 (70.7)	0.86 (0.44–1.65)	0.644		
	Four	59 (31.4)	129 (68.6)	0.77 (0.41–1.46)	0.429		
	>Four	56 (25.2)	166 (74.8)	1.05 (0.56–1.97)	0.880		
Type of family	Nuclear	116 (29.5)	277 (70.5)	1			
	Joint	46 (25.1)	137 (74.9)	1.25 (0.83–1.86)	0.277		
	Extended	13 (28.3)	33 (71.7)	1.06 (0.54–2.09)	0.860		
Religion	Hindu	105 (40.4)	155 (59.6)	1			
	Adidharma	70 (19.3)	292 (80.7)	2.83 (1.97–4.05)	<0.001	2.76 (1.79–4.26)	0.001*
Educational status	Illiterate	42 (20.5)	163 (79.5)	1.29 (0.59–2.86)	0.524		
	Primary school	101 (35.4)	184 (64.6)	0.60 (0.29–1.29)	0.196		
	Secondary school	22 (23.9)	70 (76.1)	1.06 (0.44–2.51)	0.893		
	PUC and above	10 (25.0)	30 (75.0)	1			

Socio-demographic Characteristics	Category	Attitude towards Reproductive Healthcare Services					
		Favorable n = 281 No. (%)	Unfavorable n = 340 No. (%)	C OR (95% CI)	*p* value	AOR (95% CI)	*p* value
Head of the household	Male	80 (36.5)	139 (63.5)	0.58 (0.41–0.81)	0.001	1.41 (0.95–2.10)	0.086
	Female	201 (50.0)	201 (50.0)	1			
Age	≤60 years	225 (49.1)	233 (50.9)	1			
	>60 years	56 (34.4)	107 (65.6)	1.85 (1.27–2.68)	0.001	1.81 (1.18–2.79)	0.006*
Block	Udupi	18 (22.8)	61 (77.2)	24.69 (9.55–63.77)	<0.001	11.21 (4.06–30.10)	<0.001*
	Kaup	23 (21.1)	86 (78.9)	27.24 (10.91–67.96)	<0.001	15.03 (5.71–39.29)	<0.001*
	Kundapura	85 (45.7)	101 (54.3)	8.68 (3.73–20.07)	<0.001	5.23 (2.18–12.59)	<0.001*
	Karkala	35 (52.2)	32 (47.8)	6.66 (2.64–16.78)	<0.001	7.69 (2.96–19.11)	<0.001*
	Brahmavara	58 (72.5)	22 (27.5)	2.76 (1.09–7.00)	0.032	1.59 (0.59–4.24)	0.356
	Byndoor	11 (26.2)	31 (73.8)	20.53 (7.20–58.52)	<0.001	17.99 (6.05–53.54)	<0.001*
	Hebri	51 (87.9)	7 (12.1)	1			
Total family members	Two	31 (47.7)	34 (52.3)	1			
	Three	75 (51.4)	71 (48.6)	0.86 (0.48–1.55)	0.622		
	Four	77 (41.0)	111 (59.0)	1.31 (0.74–2.32)	0.345		
	>Four	98 (44.1)	124 (55.9)	1.15 (0.66–2.01)	0.613		
Type of family	Nuclear	195 (49.7)	197 (50.3)	1			
	Joint	66 (36.1)	117 (63.9)	1.75 (1.22–2.52)	0.002	1.72 (1.16–2.66)	0.014*
	Extended	20 (43.5)	26 (56.5)	1.29 (0.70–2.38)	0.422	1.08 (0.54–2.16)	0.831
Religion	Hindu	170 (65.4)	90 (34.6)	1			
	Adidharma	111 (30.7)	250 (69.3)	4.25 (3.03–5.97)	<0.001	3.18 (2.06–4.91)	<0.001*
Educational status	Illiterate	79 (38.5)	126 (61.5)	1.60 (0.81–3.15)	0.179		
	Primary school	146 (51.4)	138 (48.6)	0.94 (0.49–1.83)	0.868		
	Secondary school	36 (39.1)	56 (60.9)	1.56 (0.73–3.29)	0.247		
	PUC and above	20 (50.0)	20 (50.0)	1			

**p* < 0.05 indicates statistically significant association*.

Religion, block and age of respondents had positive association with knowledge on services towards reproductive health, while age of respondents, block, type of family, and religion had positive association with attitudes towards the same domain.

Although on univariate analysis, all the variables except educational status were found to be significantly associated with knowledge on mental healthcare services (p < 0.05), the multivariable logistic regression analysis found that the predictors of inadequate knowledge towards mental healthcare services were residents living in Udupi (AOR = 3.86, 95% CI 1.52–9.81), Kaup (AOR = 11.37, 95% CI 4.61–28.07), Kundapura (AOR = 7.79, 95% CI 3.40–17.81), Karkala (AOR = 5.40, 95% CI 2.18–13.36) and Byndoor block (AOR = 6.26, 95% CI 2.32–16.88) and total four members in the family (AOR = 2.18, 95% CI 1.14–4.16.)

Similarly, on the univariate analysis, gender of the head of households, age, block, type of family and religion were significantly associated with unfavorable attitude towards mental healthcare services (p < 0.05). Multivariable analysis showed that the variables such as age above 60 years (AOR = 1.93, 95% CI 1.23–3.03), being residents of Udupi (AOR = 3.02, 95% CI 1.24–7.36), Kaup (AOR = 29.71, 95% CI 10.50–84.10), Kundapura (AOR = 2.62, 95% CI 1.23–5.54),Karkala (AOR = 7.99, 95% CI 3.43–18.59) and Byndoor block (AOR = 43.84, 95% CI 11.13–172.59), and following Adidharma religion (AOR = 4.10, 95% CI 2.51–6.68) were significant predictors of unfavorable attitude towards mental healthcare services ( Table 5).

**Table 5.** Association between knowledge and attitude towards healthcare services on mental health with demographic variables of the study population (n = 622).

Socio-demographic Characteristics	Category	Knowledge towards Mental Healthcare Services					
		Adequate n = 311 No.(%)	Inadequate n = 311 No. (%)	Crude OR (95% CI)	*p* value	AOR (95% CI)	*p* value
Head of the household	Male	91 (41.6)	128 (58.4)	0.59 (0.42–0.82)	0.002	1.37 (0.94–1.98)	0.100
	Female	220 (54.6)	183 (45.4)	1			
Age	≤60 years	240 (52.4)	218 (47.6)	1			
	>60 years	71 (43.3)	93 (56.7)	1.44 (1.01–2.07)	0.045	1.39 (0.93–2.09)	0.108
Block	Udupi	41 (51.2)	39 (48.8)	5.94 (2.50–14.13)	<0.001	3.86 (1.51–9.81)	0.005*
	Kaup	29 (26.6)	80 (73.4)	17.24 (7.30–40.69)	<0.001	11.37 (4.60–28.07)	<0.001*
	Kundapura	73 (39.2)	113 (60.8)	9.68 (4.34–21.58)	<0.001	7.79 (3.40–17.81)	<0.001*
	Karkala	36 (53.7)	31 (46.3)	5.38 (2.21–13.07)	<0.001	5.40 (2.18–13.36)	<0.001*
	Brahmavara	63 (78.8)	17 (21.3)	1.69 (0.67–4.22)	0.265	1.36 (0.52–3.54)	0.523
	Byndoor	19 (45.2)	23 (54.8)	7.57 (2.89–19.81)	<0.001	6.26 (2.32–16.88)	<0.001*
	Hebri	50 (86.2)	8 (13.8)	1			
Total family members	Two	44 (67.7)	21 (32.3)	1			
	Three	89 (60.5)	58 (39.5)	1.37 (0.73–2.53)	0.322	1.18 (0.61–2.30)	0.631
	Four	85 (45.2)	103 (54.8)	2.54 (1.40–4.60)	0.002	2.18 (1.14–4.16)	0.018*
	>Four	93 (41.9)	129 (58.1)	2.91 (1.62–5.21)	<0.001	2.03 (1.01–4.09)	0.047*
Type of family	Nuclear	213 (54.2)	180 (45.8)	1			
	Joint	80 (43.7)	103 (56.3)	1.52 (1.07–2.17)	0.019	1.09 (0.67–1.76)	0.737
	Extended	18 (39.1)	28 (60.9)	1.84 (0.99–3.44)	0.055	1.02 (0.48–2.19)	0.955
Religion	Hindu	155 (59.6)	105 (40.4)	1			
	Adidharma	156 (43.1)	206 (56.9)	1.95 (1.41–2.69)	<0.001	1.50 (0.99–2.29)	0.058
Educational status	Illiterate	98 (47.8)	107 (52.2)	1.64 (0.82–3.26)	0.161		
	Primary school	146 (51.2)	139 (48.8)	1.43 (0.73–2.80)	0.300		
	Secondary school	43 (46.7)	49 (53.3)	1.71 (0.81–3.63)	0.163		
	PUC and above	24 (60.0)	16 (40.0)	1			

Socio-demographic Characteristics	Category	Attitude on Mental Healthcare Services					
		Favourable n = 246 No. (%)	Unfavourable n = 376 No. (%)	UOR (95% CI)	*p* value	AOR (95% CI)	*p* value
Head of the household	Male	74 (33.8)	145 (66.2)	0.69 (0.49–0.97)	0.030	1.07 (0.70–164)	0.740
	Female	172 (42.7)	231 (57.3)	1			
Age	≤60 years	198 (43.2)	260 (56.8)	1			
	>60 years	48 (29.3)	116 (70.7)	1.84 (1.25–2.70)	0.002	1.93 (1.23–3.03)	0.004*
Block	Udupi	25 (31.3)	55 (68.8)	8.43 (3.82–18.62)	<0.001	3.02 (1.24–7.36)	0.015*
	Kaup	7 (6.4)	102 (93.6)	55.86 (20.65–151.08)	<0.001	29.71 (10.50–84.10)	<0.001*
	Kundapura	83 (44.6)	103 (55.4)	4.76 (2.37–9.56)	<0.001	2.61 (1.23–5.54)	0.012*
	Karkala	24 (35.8)	43 (64.2)	6.87 (3.06–15.41)	<0.001	7.99 (3.43–18.59)	<0.001*
	Brahmavara	58 (72.5)	22 (27.5)	1.45 (0.65–3.25)	0.361	0.69 (0.29–1.67)	0.415
	Byndoor	3 (7.1)	39 (92.9)	49.83 (13.11–189.39)	<0.001	43.83 (11.13–172.59)	<0.001*
	Hebri	46 (79.3)	12 (20.7)	1			
Total family members	Two	30 (46.2)	35 (53.8)	1			
	Three	67 (45.6)	80 (54.4)	1.02 (0.57–1.84)	0.938		
	Four	74 (39.4)	114 (60.6)	1.32 (0.75–2.33)	0.338		
	> Four	75 (33.8)	147 (66.2)	1.68 (0.96–2.95)	0.070		
Type of family	Nuclear	172 (43.8)	221 (56.2)	1			
	Joint	59 (32.2)	124 (67.8)	1.64 (1.13–2.36)	0.009	1.50 (0.95–2.36)	0.083
	Extended	15 (32.6)	31 (67.4)	1.61 (0.84–3.07)	0.150	1.27 (0.57–2.79)	0.560
Religion	Hindu	148 (56.9)	112 (43.1)	1			
	Adidharma	98 (27.1)	264 (72.9)	3.56 (2.54–4.99)	<0.001	4.10 (2.51–6.68)	<0.001*
Educational status	Illiterate	73 (35.6)	132 (64.4)	1.09 (0.54–2.19)	0.820		
	Primary school	125 (43.9)	160 (56.1)	0.77 (0.39–1.52)	0.448		
	Secondary school	33 (35.9)	59 (64.1)	1.07 (0.50–2.31)	0.858		
	PUC and above	15 (37.5)	25 (62.5)	1			

**p* < 0.05 indicates statistically significant association*.

Block and a greater number of family members had positive association with knowledge on services towards mental health, while taluk and religion had positive association with attitudes towards the same domain.

## Discussion

The present study included 622 individuals belonging to the Koraga tribe living across seven blocks of the Udupi district. The study findings showed that the tribal population have poor knowledge (62.2%) and unfavourable attitude (50.2%) towards healthcare services on communicable diseases, and this aligns with previous reports that supports high prevalence of communicable diseases among various tribes across India. ^20– 22^ Our findings are supported by an Australian study by Rheault et al., which showed that 59 per cent of the tribal population in the study area had low literacy rate which hindered their knowledge and reduced the access to healthcare services. ^23^ Approximately 93% of the tribal population in India resides in rural and remote areas, which significantly limits their access to formal education and schooling opportunities. ^24^ Our study also revealed notable gender disparities in knowledge and attitudes towards healthcare services for communicable diseases, with 68.5% of males and 58.8% of females demonstrating poor knowledge. Interestingly, attitudes were similarly unfavourable among both genders, with 49.3% of males and 50.6% of females reporting negative perceptions. Age emerged as a significant factor, with 78% of respondents aged over 60 years exhibiting poor knowledge, and 60.4% of the same age group had unfavourable attitudes, indicating a strong association between advancing age and limited awareness and receptiveness to healthcare services (p < 0.05). Geographic disparities were evident, with residents of Udupi and Kaup taluks exhibiting markedly higher proportions of poor knowledge (80%) and unfavourable attitudes (70.6%) toward healthcare services, both statistically significant (p < 0.05). Furthermore, individuals identifying with the Adidharma religion and those who were illiterate were significantly more likely to have both poor knowledge (<0.001) and unfavourable attitudes (<0.001) towards communicable disease-related healthcare services (p < 0.05), underscoring the influence of socio-cultural and educational factors on health perceptions. An Acquired Immuno Deficiency Syndrome (AIDS) awareness study conducted in Karnataka, a southern state of India by Eknath Naik et al. supports our findings in some respects. The study revealed that only 22% of adults had ever heard of AIDS, and just 18% knew about its modes of transmission. Furthermore, only 5% were aware of the link between STDs and AIDS. Notably, AIDS awareness among women was significantly lower than among men (14% vs. 30%), which contrasts with our findings, where females demonstrated better knowledge than males (41.2% vs. 39.5%). ^25^ However, a study conducted among a tribal community in Assam by Phukan presents contrasting findings which demonstrated a high level of awareness regarding malaria symptoms (97.3%), treatment options (94%), preventive measures (88%), and diagnostic methods (68%) among the respondents. Despite this, their knowledge about the availability of services from Fever Treatment Depots (FTDs) and Drug Distribution Centres (DDCs) was notably limited. ^26^ Our study is supported by research conducted in Maharashtra by Rajvanshi et al, which revealed poor knowledge and an unfavourable attitude towards malaria treatment among a tribal population. The study found that, 64.4% of respondents were illiterate, and only 20.6% were aware of the causative factors of malaria, despite 45% of household heads having received some form of health education from various sources. ^27^ Similarly, a study conducted among a tribal population in Maharashtra found that only 19% of participants were aware of the transmission routes of sexually transmitted diseases (STDs), and 30% relied on home remedies as a treatment option. ^28^ The variations in knowledge levels among tribal communities can be attributed to cultural distinctions and traditional practices, further influenced by multiple factors such as geographical terrain, remoteness from mainstream society, and the unique ancestral customs upheld by each group. ^28, 29^

The findings of our research revealed that 71.9% of respondents had poor knowledge, and 54.8% exhibited an unfavourable attitude towards reproductive healthcare services. Notably, majority of the males (78.1%) and females (68.5%) demonstrated poor knowledge. Statistically significant associations (p < 0.05) were observed among participants aged above 60 years (84.8%), residents of Kaup taluk (89%), and individuals belonging to the *Adidharma* tribal group (80.7%), households with more than four members (74.8%), extended family structures (71.7%), and illiteracy (79.5%) were also identified as significant demographic factors associated with poor knowledge among the respondents. Similarly, attitude scores were also more likely to be unfavourable among males (63.5%), individuals aged above 60 years (65.5%), residents of Udupi taluk (77.2%), those living in joint families (63.9%), and members of the *Adidharma* religion (69.3%). These associations were statistically significant (p < 0.05). Factors such as large family size (35.7%) and low literacy (33.0%) may have contributed to an unfavourable attitude towards reproductive and child health. Our findings are consistent with the qualitative research done by Bharathi et al, in Uttarakhand which found that the Tharu tribe and the Buksa tribe had very poor knowledge and attitude towards the maternal and childcare provided by the healthcare system. ^30^ Another qualitative study from Bangladesh among Garo tribe also supports our findings, which elicited socio-economic factors, culture-based attitude and practices, family dynamics, husband’s knowledge and personal dispositions to play a significant role in the attitude towards reproductive healthcare services. The research found that most of these factors are unfavourable for the Garo tribe to access and avail reproductive healthcare services. Research evidence among tribal women in north India, also are consistent with our findings, that the favourable attitude is influenced by many demographic variables and educational status. The study showed that the utilization of antenatal services among ST women varied from about four per cent in Madhya Pradesh and Rajasthan to 10–14 per cent in Chhattisgarh and Odisha. Utilization was higher among those women with nine or more years of schooling (15–28%) and those women who visited health facility for pregnancy confirmation test (9–27%). ^33, 34^ Similarly, a study conducted in the coastal Karnataka found that although 82.9 per cent of tribal women had heard of cervical cancer, only 2.3 per cent knew it could be detected early, and none had undergone screening, which could be attributed to lack of awareness and poor accessibility to healthcare services. ^35^ Another study in Madhya Pradesh reported significant lack of awareness about cervical cancer and its link to Human Papilloma Virus (HPV) infection among tribal women, leading to negative attitudes towards screening services. ^36^ Contrasting to our findings, a paper published from Canada reported that there is a positive attitude among the indigenous women towards reproductive healthcare services. ^37^ In contrast Aswathi et al, argues that improving knowledge and attitude can enhance the accessibility and utilization of reproductive healthcare services, with their results showing 74.2 per cent of study participants being aware that cervical cancer could be detected early by a screening test. Majority of respondents (89.2%) did not have awareness on risk factors for cervical cancer (p < 0.05). ^38^

Poor knowledge (50%) and unfavourable attitude (60.5%) towards mental health services among the Koraga community are consistent with studies from India among other tribal populations. Males had comparatively more knowledge (58.0%) than females (45.4%) and the knowledge was found to be adequate among respondents aged above 60 years (56.7%). Additionally, those from extended family (60.9%), *Adidharma* religion (56.9%), aged above 60 years (70.7%) and those with low literacy (52.2%) also showed poor knowledge regarding mental health services. Similarly, males (66.2%), females (57.3%), joint family (67.8%), *Adidharma* religion (72.9%, p < 0.05) and illiteracy (64.4%) were also having an unfavourable attitude. Significantly higher proportion of participants from Kaup (93.6%), Udupi (68.8%), Karkala (64.2%) and Kundapura blocks (55.4%), had (p < 0.05) unfavourable attitude. A systematic review by Bakhla et al. on mental health problems among tribes, showed that the prevalence of mental health disorders varied across the studies with depression ranging from three per cent to 43 per cent, anxiety up to eight per cent, mania from 0.04 per cent to 0.39 per cent, schizophrenia from 0.07 per cent to 0.52 per cent, intellectual disability from 0.1 per cent to 0.6 per cent, epilepsy from 0.2 per cent to 1.03 per cent, and dementia or cognitive impairment up to 42.92 per cent. A meta-analysis of 15 studies on depression among tribal population in India found that the pooled prevalence to be 14 per cent (with a confidence interval of 8% to 22%). ^39^ A mixed method study in Odisha found that the low educational level, Hindu religion, higher age, and female gender ( *p* < 0.05) were significantly associated with attitudes towards mental healthcare facilities among people in the remote and rural communities. It was also found that female respondents had more negative attitudes than male respondents. ^40^ Jena. S et al & Ali. T et al examined the attitudes and practices of tribal communities towards mental health in Jharkhand and other catchment areas. The results showed that over 60% of people believed in supernatural causes of mental illness and do not seek healthcare services. ^41, 42^ Contrastingly Noronha et al, in her research found that the majority (80%) of the Koraga tribal participants had good knowledge regarding mental health, and 68% of them had a favourable attitude towards mentally ill individuals. It was found that 79.2% of the participants had a favourable attitude. ^43^ Our findings are supported by another study by Deb Roy A et al.which reiterates the importance of mental health education and literacy to enhance the knowledge and attitude of the population to improve the utilization of health care services. ^44^ Additionally, findings from recent ethnographic research conducted among the PVTG emphasised the need for the implementation of effective administrative policies and well-coordinated healthcare activities. Such measures would significantly increase the likelihood of successful outcomes by empowering these communities to adopt and effectively utilize the resources available to them, thereby improving their outlook and access to healthcare services. ^45^

### Limitations

The study was conducted among Koraga tribal community, the only PVTG in Udupi district and hence it lacks the scope for generalization of results to a larger population. Considering other PVTGs and conducting a comparative study across various states of India would be helpful to assess additional barriers to healthcare access. Notably, this study is unique in being the first to explore the knowledge and attitude towards healthcare services among the Koraga tribe of Karnataka. We have conducted this study considering possible bias related to the subjects, samples, (being tribal population) researchers, and methodology and by conducting this study in a very scientific and systematic manner, we have strived the best to eliminate the chances of biases.

### Suggestions for future research

Studies can be focused on other PVTGs elsewhere in India with objectives of comparing the knowledge, attitude and practice on healthcare services which can be compared with general population also. The present research has highlighted several barriers which hinder the accessibility of healthcare services for tribal populations that can be overcome through coordinated education and health literacy among tribal populations. ^46– 49^ Future research can utilize larger samples to make the results generalizable and such studies will be able to bring out hidden facts about tribal health which in turn might help the healthcare system to develop more fortified and structured programs for the improvement of tribal health, aligning with various Sustainable Development Goals. Qualitative studies can help to understand the underlying cultural, social, and systemic factors that influence healthcare-related knowledge, attitudes, and practices among this PVTG.

## Conclusion

Although a range of healthcare services is available to tribal populations in India, their utilization remains suboptimal. The findings of this study indicate that the Koraga tribe exhibits limited knowledge and an overall unfavourable attitude toward healthcare services and their utilization. Specifically, the tribe demonstrated inadequate awareness and negative perceptions across three critical domains: communicable diseases, reproductive health, and mental health. While their knowledge and attitudes regarding communicable and reproductive health were found to be at a moderate level, their understanding of mental illness and related healthcare services was notably poor, accompanied by markedly negative attitudes.

These deficits in knowledge and attitude appear to act as significant barriers to accessing and effectively utilizing both public and private healthcare services. The study also found that factors such as cultural and traditional health beliefs, geographic isolation and low educational attainment, were significantly associated with their unfavourable attitudes toward the healthcare system and services.

The findings of this study provide important directions for future research PVTGs, especially concerning their cultural beliefs, existing practices, knowledge gaps, and potential interventions. These insights may assist healthcare administrators and policymakers in designing culturally sensitive health education initiatives and motivational programs aimed at improving healthcare access and utilization among tribal populations.

### Data protection issues

This cross- sectional study was conducted among a Particularly Vulnerable Tribal Group (PVTG), and the knowledge questionnaire and attitude scale which were used in this research is shared in the extended data. Any further dataset such as score sheet can be available on request from the corresponding author, mentioning the specific reason for availing it.

## Ethical and consent statement

Permission was obtained from the Institutional Ethics Committee (KMC-KH IEC) of Kasturba Medical College, Manipal, Manipal Academy of Higher Education. (IEC No: 778/2019) Approved on 19 November 2019). A written informed consent was received from all participants. This research was conducted in accordance with the Declaration of Helsinki.

## Data Availability

Since the study was conducted among a socio-cultural and economically vulnerable group, and their stakeholders, the authors have taken precautions as per the guidelines by Declarations of Helsinki to protect the confidentiality and privacy of the participants and the data is provided based on that. The readers may contact the corresponding author for further information on this research on ranjitha.shetty@manipal.edu 1. Figshare. Extended Data (Questionnaire, Participant information Sheet & Informed Consent). https://doi.org/10.6084/m9.figshare.31109209 ^51^
This project contains the following underlying data: • Extended data on (a) Interviewer administered Knowledge Questionnaire and Attitude Scale (b) Participant Information Sheet (c) Informed Consent form (d) Knowledge and Attitude Score Sheet Data is available under the terms of the CC BY 4.0. 2. Figshare. Extended Data on Knowledge and Attitude Scores. https://doi.org/10.6084/m9.figshare.31055596 ^52^
This project contains the following underlying data: • Tables Data is available under the terms of the CC BY 4.0. The guidelines for reporting this study are available in Figshare: STROBE Statement—Checklist of items that should be included in reports of ***cross-sectional studies***. https://doi.org/10.6084/m9.figshare.30996508 ^50^ The data are available under the terms of the Creative Commons Attribution 4.0 International license (CC-BY 4.0).
