# Supplementary material for: Knowledge and Attitudes Toward Healthcare Services Among a Particularly Vulnerable Tribal Group (PVTG) in Coastal Karnataka, India: Findings from a Community-Based Cross-Sectional Survey
Source: F1000Res. 2026 Jun 25;15:542. Originally published 2026 Apr 16. [Version 2] doi: 10.12688/f1000research.176835.2 (PMC13554813; doi:10.12688/f1000research.176835.2)
Supplement: Peer review history

# Reviewer report: Reviewer response for version 2

**DOI:** 10.5256/f1000research.204378.r498488

**Published:** 2026-07-23

**Authors:** Santhosh Gugulothu

**Affiliations:** 1 Centre for Economic and Social Studies, Hyderabad, Telangana, India

**Summary**

 The study assessed the knowledge and attitudes towards healthcare services among 622 adult members of the Koraga, a Particularly Vulnerable Tribal Group (PVTG), living across seven blocks of Udupi district in coastal Karnataka. The study uses a community-based cross-sectional design and multistage cluster sampling, the authors examined awareness and attitudes related to communicable diseases, reproductive health, and mental health services. The findings showed that a large proportion of participants had inadequate knowledge and unfavorable attitudes across all three domains, particularly for reproductive and mental health. Older age, low educational status, geographical location, religion, and family characteristics were significantly associated with poorer knowledge and attitudes. The authors conclude that culturally appropriate health literacy programmes and targeted interventions are needed to improve healthcare awareness and utilization among the Koraga community.

**Major Comments**

 This manuscript addresses an important and under-researched topic by examining knowledge and attitudes towards healthcare services among the Koraga tribe.  The study is methodologically rigorous, with an adequate sample size (622 participants) and utilizes appropriate statistical analyses. The findings provide useful baseline information on the healthcare-related knowledge and attitudes of this community.

 However, I have several conceptual concerns regarding the interpretation and contribution of the findings that should be addressed.

**1. Novelty and contribution of the study need to be clarified.**

 Although the study focuses on a PVTG, the manuscript does not clearly explain what new knowledge it contributes beyond existing literature. Previous studies have consistently demonstrated that tribal and particularly PVTG communities experience poor health outcomes due to social exclusion, poverty, low educational attainment, geographical isolation, and inadequate healthcare access. The present findings that the Koraga community has limited knowledge and unfavorable attitudes towards healthcare services, are therefore largely expected.

  

- The authors should explicitly articulate, what specific knowledge gap this study fill?
- How do the findings of this study advance our understanding beyond confirming what is already well-known?
- How can these findings inform policy differently from previous tribal health research?

**2. The conclusions regarding healthcare utilisation are not adequately supported.**

 The study concludes that healthcare services are available but are underutilized because of poor knowledge and unfavorable attitudes. This interpretation seems simplistic and is not sufficiently supported by the presented data. The study itself acknowledges several structural and social barriers which includes geographical isolation, remote forest settlements, dependence on public transport, distance from health facilities, low educational attainment, and socio-cultural practices. These factors are well-established determinants of healthcare utilisation. The limited utilisation may reflect problems of accessibility, affordability, and acceptability of services. Therefore, we cannot attribute the underutilisation mainly to community-level deficits.

**3. The study emphasises lack of awareness but does not sufficiently explore the context behind this finding.**

 Throughout the manuscript, inadequate knowledge is treated as the principal problem. However, for a PVTG living in geographically isolated and socially marginalised settings, limited awareness is itself an expected consequence of historical deprivation. Instead of stating that awareness is low, the discussion should focus more critically on why awareness remains poor.

 Structural determinants should be emphasised more, for instance educational disadvantage, historical marginalisation of the community, there may be a limited health communication and linguistic barriers or cultural beliefs. So, understanding these underlying aspects would make discussion more meaningful.

**4. Important dimensions of healthcare access are missing.**

 The study focuses on knowledge and attitudes but provides very little information regarding actual access to healthcare services. For example, the research should have focused on awareness of government tribal health schemes and their utilisation, perceived quality of healthcare, trust in healthcare providers (as most PVTGs in India relays on traditional healthcare medicines), the study does not consider their financial barriers and discrimination within healthcare settings.

 Without observing these factors, concluding that the community is not utilising healthcare primarily because of poor knowledge may be misrepresentative.

**5. Mental health findings require more contextual interpretation.**

 The study reports poor knowledge and unfavourable attitudes towards mental health services. However, the discussion largely interprets this as a deficit in awareness. Given the remoteness and socio-cultural context of the Koraga community, the authors should acknowledge that concepts of mental illness or wellbeing may differ substantially from standard definitions.

 Therefore, it is important to discuss the indigenous understandings of mental illness, their traditional healing practices and availability of mental health services.

**7. Recommendations should extend beyond health education.**

 The manuscript recommends health literacy programmes. Although, health education is important, the findings also indicate the need for broader interventions such as improving physical accessibility of healthcare, strengthening outreach through ASHAs and tribal health workers, culturally appropriate health communication, addressing transportation barriers, and strengthening implementation of tribal welfare schemes,

**Minor Comments**

- The discussion is lengthy and could be reorganized by reducing repetitive comparisons with previous studies.
- The manuscript would benefit from a clearer conceptual framework where it can link structural determinants, knowledge, attitudes, and healthcare utilisation.

**Overall Recommendation**

 This is a well-designed community-based study which addresses an important public health issue among a Particularly Vulnerable Tribal Group. The sampling strategy, instrument validation, and statistical analyses are appropriate. However, the manuscript’s primary weakness lies in the interpretation of the findings. The discussion and conclusions place considerable emphasis on deficits in knowledge and attitudes and gives insufficient attention to the broader structural, social, and health system factors which shape healthcare utilisation among PVTGs. Clarifying the study’s novelty, contextualising the findings within the realities of tribal marginalisation, and revising the conclusions accordingly would strengthen the manuscript.

Is the work clearly and accurately presented and does it cite the current literature?

Yes

If applicable, is the statistical analysis and its interpretation appropriate?

I cannot comment. A qualified statistician is required.

Are all the source data underlying the results available to ensure full reproducibility?

Partly

Is the study design appropriate and is the work technically sound?

Yes

Are the conclusions drawn adequately supported by the results?

Partly

Are sufficient details of methods and analysis provided to allow replication by others?

Yes

Reviewer Expertise:

Sociology, Social inequality, Health, Education, and Migration among Indigenous and Tribal Studies in India. Denotified Tribal communities.

I confirm that I have read this submission and believe that I have an appropriate level of expertise to confirm that it is of an acceptable scientific standard, however I have significant reservations, as outlined above.

# Reviewer report: Reviewer response for version 2

**DOI:** 10.5256/f1000research.204378.r498493

**Published:** 2026-07-21

**Authors:** Lalitha Rani Chellappa

**Affiliations:** 1 Saveetha Dental College, Saveetha Institute of Medical and Technical Sciences, Chennai, Tamil Nadu, India

- **Questionnaire not described sufficiently**
  - Although the number of knowledge and attitude items is reported, the actual questionnaire is not included in the manuscript (only mentioned as extended data).
  - The rationale for item development should be explained more clearly.
- **Median split for knowledge and attitude**
  - Dichotomising continuous/ordinal scores using the median can reduce statistical power and lead to loss of information.
  - The authors should justify why the median was chosen instead of validated cut-offs or analysing the scores as continuous variables.
- **Cluster sampling not accounted for in analysis**
  - The study used multistage cluster sampling, but the logistic regression appears to treat observations as independent.
  - Please clarify whether clustering at village/block level was adjusted for (e.g., robust standard errors, survey-weighted analysis or multilevel modelling). If not, discuss this as a limitation.
- **Potential social desirability bias**
  - Face-to-face interviews among tribal populations can influence responses.
  - This should be acknowledged in the limitations.
- **Discussion**
  - The discussion mainly compares prevalence values.
  - It would benefit from deeper interpretation of *why* older age, education and religion were associated with knowledge and attitudes, with reference to behavioural or health service utilisation theories.

Is the work clearly and accurately presented and does it cite the current literature?

Partly

If applicable, is the statistical analysis and its interpretation appropriate?

Partly

Are all the source data underlying the results available to ensure full reproducibility?

Partly

Is the study design appropriate and is the work technically sound?

Yes

Are the conclusions drawn adequately supported by the results?

Yes

Are sufficient details of methods and analysis provided to allow replication by others?

Yes

Reviewer Expertise:

public health, epidemiology, systematic reviews, and biostatistics

I confirm that I have read this submission and believe that I have an appropriate level of expertise to confirm that it is of an acceptable scientific standard, however I have significant reservations, as outlined above.

# Reviewer report: Reviewer response for version 1

**DOI:** 10.5256/f1000research.194947.r476278

**Published:** 2026-05-11

**Authors:** Dr Jayashree Gothankar

**Affiliations:** 1 Bharati Vidyapeeth Deemed University Medical College, Pune, India

The validation and pre-testing of research tools need to be written.  

 Statistical analysis: It is preferred to use the prevalence ratio instead of the odds ratio. Since the outcome is common, here, the odds ratio can exaggerate the association. Thus, the prevalence ratio and the adjusted prevalence ratio with 95% confidence intervals may provide a more accurate measure of the association between various characteristics and the knowledge and attitude of the tribal population.

 Regarding the limitations, kindly elaborate on the statement “We have conducted this study considering possible bias related to the subjects, samples (being a tribal population), and researchers.”

Is the work clearly and accurately presented and does it cite the current literature?

Yes

If applicable, is the statistical analysis and its interpretation appropriate?

Partly

Are all the source data underlying the results available to ensure full reproducibility?

Yes

Is the study design appropriate and is the work technically sound?

Yes

Are the conclusions drawn adequately supported by the results?

Yes

Are sufficient details of methods and analysis provided to allow replication by others?

Partly

Reviewer Expertise:

Implementation research, public health, epidemiology

I confirm that I have read this submission and believe that I have an appropriate level of expertise to confirm that it is of an acceptable scientific standard.

# Response

**Published:** 2026-06-19

**Authors:** Dr Ranjitha S Shetty

**Affiliations:** Community Medicine, Manipal Academy of Higher Education, Manipal, Karnataka, India

**1.The validation and pre-testing of research tools need to be written.**

 Response: Thank you for noting this. Validation and pretesting were done prior to IEC approval. However, we missed adding details in the manuscript. Please note that we have added the details of validation and pre-testing under methods section now.

**2. Statistical analysis: It is preferred to use the prevalence ratio instead of the odds ratio. Since the outcome is common, here, the odds ratio can exaggerate the association. Thus, the prevalence ratio and the adjusted prevalence ratio with 95% confidence intervals may provide a more accurate measure of the association between various characteristics and the knowledge and attitude of the tribal population.**

 Response: Thank you for providing this valuable suggestion. We sincerely appreciate the reviewer's expertise in drawing our attention to this important methodological consideration, and we are glad to have the opportunity to clarify our analytical approach. We acknowledge that prevalence ratios are often preferred as a more direct and interpretable measure of association in cross-sectional studies. In the present analysis, logistic regression was used to model binary outcomes, as it is a well-established and robust method that provides stable estimates.

**3. Regarding the limitations, kindly elaborate on the statement “We have conducted this study considering possible bias related to the subjects, samples (being a tribal population), and researchers.”**

 Response: Thank you for the comment. We have clarified the statement as follows: Potential biases related to participants, sampling, researchers, and methodology were carefully considered. Although the researcher was not from the tribal community, the involvement of tribal leaders and local health workers familiar with the participants’ language and culture helped minimize contextual bias.

# Reviewer report: Reviewer response for version 1

**DOI:** 10.5256/f1000research.194947.r476269

**Published:** 2026-05-05

**Authors:** Prof. (Dr.) Roumi Deb

**Affiliations:** 1 Department of Anthropology, AISS, Amity University, Noida, Uttar Pradesh, India

1. Summary of the article-

 This study is a community-based cross-sectional survey conducted among 622 adults from the Koraga tribe, a Particularly Vulnerable Tribal Group (PVTG) in Udupi district, Karnataka, India.  The objective was to assess knowledge and attitudes toward healthcare services across three domains- communicable diseases, reproductive health, and mental health. Using multistage cluster sampling and structured interviews, the authors found that a large proportion of participants had poor knowledge (up to 71.9%) and unfavourable attitudes (up to 60.5%) toward healthcare services. Sociodemographic factors such as age, education, religion, and geographic location were significantly associated with knowledge and attitudes.

 The authors conclude that targeted health literacy interventions are needed to improve healthcare utilization among this population.

 2. Comments linked to questions above-

 A. Clarity of presentation and use of literature - Partly

 The manuscript is generally well-structured (clear abstract, methods, results, discussion). Background section provides context on tribal health disparities. Relevant studies are cited to support findings and comparisons.

 Though, there is limited emphasis on recent (last 5 years) high-impact literature. Literature review lacks clear identification of knowledge gaps specific to this population.

 It is recommended to include more recent and regionally comparable studies. Also, clearly articulate how this study adds new knowledge beyond existing evidence.

 B. Study design and technical soundness - Yes

 Appropriate cross-sectional design has been used for assessing knowledge and attitudes. Use of multistage cluster sampling with PPS is methodologically sound. Adequate sample size calculation and justification is provided. Ethical approval and informed consent procedures are well described.

 C. Methods and reproducibility - Partly

 Sampling method and study setting are described in detail. The questionnaire structure and scoring system are explained. Statistical methods are also outlined. However, limited detail has been provided on validation process (e.g., reliability coefficients not reported), no discussion of potential biases (interviewer bias, social desirability bias).

 It is recommended to provide psychometric properties (Cronbach’s alpha, validity metrics) and include bias mitigation strategies.

 D. Statistical analysis - Partly

 Use of logistic regression (univariate and multivariable) is appropriate. Reporting of AORs with 95% CIs is correct. However, there is no mention of model diagnostics (goodness-of-fit, multicollinearity), handling of confounders.

 It is recommended to include model fit statistics (e.g., Hosmer–Lemeshow test) and confounding variables.

 E. Data availability and reproducibility - Yes

 Extended data is shared via Figshare.

 F. Conclusions supported by results - Yes

 Conclusions align with findings (poor knowledge and attitudes). Policy implications (health literacy interventions) are reasonable.

 3. Essential Revisions (Must be addressed)-

 To ensure scientific soundness, the authors should:

 3.1 Expand statistical reporting- Include model diagnostics and clarify confounding variables.

 3.2 Improve methodological transparency- Provide validation details and bias control measures.

 3.3 Strengthen literature review- Include recent studies and better synthesis. 

 The study is important and methodologically sound, but requires minor revisions, particularly in statistical reporting and literature review, before it can be considered fully robust for indexing.

Is the work clearly and accurately presented and does it cite the current literature?

Partly

If applicable, is the statistical analysis and its interpretation appropriate?

Partly

Are all the source data underlying the results available to ensure full reproducibility?

Yes

Is the study design appropriate and is the work technically sound?

Yes

Are the conclusions drawn adequately supported by the results?

Yes

Are sufficient details of methods and analysis provided to allow replication by others?

Partly

Reviewer Expertise:

Public Health, Bio-social Anthropology, Demography, Population studies, Population Genetics, Reproductive health, Maternal and Child heath, studies on youth

I confirm that I have read this submission and believe that I have an appropriate level of expertise to confirm that it is of an acceptable scientific standard, however I have significant reservations, as outlined above.

# Response

**Published:** 2026-06-19

**Authors:** Dr Ranjitha S Shetty

**Affiliations:** Community Medicine, Manipal Academy of Higher Education, Manipal, Karnataka, India

**1: There is limited emphasis on recent (last 5 years) high-impact literature. Literature review lacks clear identification of knowledge gaps specific to this population. It is recommended to include more recent and regionally comparable studies.**

 Response: Thank you for the comment. We have revised the review of literature to add recent studies done among Koraga and other regional tribal studies.

**2: Clearly articulate how this study adds new knowledge beyond existing evidence.**

 Response: The new knowledge that this study would add to the scientific field has been incorporated into the manuscript.

**3: Limited detail has been provided on validation process (e.g., reliability coefficients not reported), no discussion of potential biases (interviewer bias, social desirability bias). It is recommended to provide psychometric properties (Cronbach’s alpha, validity metrics) and include bias mitigation strategies.**

 Response: The instruments used in the study underwent content validation by a panel of seven experts comprising academicians and researchers with expertise in community medicine, reproductive health, and mental health. Public health experts and statisticians also evaluated the tools for relevance, clarity, and comprehensiveness. The overall Content Validity Index (CVI) was 0.82, demonstrating good content validity of the instrument. Reliability test of the tool was done by split half method for knowledge Questionnaire (0.84) and Cronbach’s Alpha for attitude scale (0.92). A pilot study was conducted among 60 participants in Udupi taluk and found feasible, and the same village and settlements were excluded from the main study.

**4. There is no mention of model diagnostics (goodness-of-fit, multicollinearity), handling of confounders. It is recommended to include model fit statistics (e.g., Hosmer–Lemeshow test) and confounding variables.**

 Response:  Multicollinearity was assessed using the Variance Inflation Factor (VIF), with all variables demonstrating VIF < 2. Model fit was evaluated using the Hosmer–Lameshow goodness-of-fit test. The results of Hosmer–Lameshow test for goodness-of-fit are now reported in the paper.
